# Deletion of immunomodulator C6 from vaccinia virus strain Western Reserve enhances virus immunogenicity and vaccine efficacy

**DOI:** 10.1099/vir.0.049700-0

**Published:** 2013-05

**Authors:** Rebecca P. Sumner, Hongwei Ren, Geoffrey L. Smith

**Affiliations:** Department of Pathology, University of Cambridge, Tennis Court Road, Cambridge CB2 1QP, UK

## Abstract

Vectors based on vaccinia virus (VACV), the vaccine used to eradicate smallpox, are currently popular candidates for the vaccination against numerous infectious diseases including malaria and AIDS. Although VACV induces robust cellular and humoral responses, enhancing the safety and efficacy of these vectors remains an important area of research. Here, we describe the enhanced immunogenicity of a recombinant VACV Western Reserve (WR) strain lacking the immunomodulatory protein C6 (vΔC6). Intradermal infection of mice with vΔC6 was shown previously to induce smaller lesions, indicating viral attenuation, and this was confirmed here using a different inoculation dose. In addition, data presented show that vaccination with vΔC6 provided better protection against challenge with a lethal dose of VACV WR, indicating this virus is a better vaccine. Increased protection was not due to improved humoral responses, but instead enhanced cytotoxic activity of T-cells 1 month post-inoculation in the spleens of vΔC6-vaccinated mice.

*Vaccinia virus* (VACV) is a member of the family *Poxviridae*, and is well known as the vaccine used for the eradication of smallpox (Fenner *et al.*, 1988). VACV, like other poxviruses, is a large dsDNA virus that replicates in the cytoplasm of infected cells (Moss, 2007). Despite the eradication of smallpox, VACV is still studied intensively: as a tool for investigating host–pathogen interactions and as a vector for vaccine development. Recombinant VACVs were developed in the early 1980s (Mackett *et al.*, 1982; Panicali & Paoletti, 1982) and are able to induce a strong host immune response, including both humoral (Panicali *et al.*, 1983; Smith *et al.*, 1983a, b) and cellular responses (Bennink *et al.*, 1984) and activating both innate and adaptive components (Delaloye *et al.*, 2009; Moss, 2011). The VACV strains used to eradicate smallpox caused some vaccine-related complications (Lane *et al.*, 1969) and therefore more attenuated strains, such as the highly attenuated modified vaccinia virus Ankara (MVA), are being tested for vaccination against several infectious diseases including human immunodeficiency virus (HIV) and malaria (Gómez *et al.*, 2008). Despite the loss of large sections of DNA following serial passage in chicken embryonic fibroblast cells (Meyer *et al.*, 1991), the MVA genome still contains some immunomodulatory genes (Blanchard *et al.*, 1998), the removal of which may enhance the immunogenicity of this vector.

VACV encodes around 200 genes, approximately half of which play essential roles in virus replication, morphogenesis and spread. The other half of the genome is made up of non-essential genes, many of which have been characterized to have roles in virus–host interactions including host tropism and evasion of immunity (Upton *et al.*, 2003; Gubser *et al.*, 2004; Lefkowitz *et al.*, 2006). Removal of immune modulatory genes might lead to viral attenuation, and hence enhanced safety. Equally, the removal of genes that dampen the immune response may have a positive impact on immunogenicity. This has already been demonstrated with VACVs lacking several proteins that are secreted from the infected cell such as the chemokine-binding protein A41 (Clark *et al.*, 2006), the IL-1β receptor B15 (Staib *et al.*, 2005; Cottingham *et al.*, 2008), the interferon (IFN) decoy receptors B18 and B8 (Gómez *et al.*, 2012) and the IL-18-binding protein C12 (Reading *et al.*, 2003b; Falivene *et al.*, 2012). Loss of intracellular proteins A35 (Rehm & Roper, 2011), the steroid biosynthetic enzyme 3-β-hydroxysteroid dehydrogenase (Moore & Smith, 1992; Reading *et al.*, 2003a) and protein C6 (García-Arriaza *et al.*, 2011) have all increased antigen-specific immune responses.

VACV WR protein C6 is a non-essential protein that is expressed early during infection (Assarsson *et al.*, 2008; García-Arriaza *et al.*, 2011; Unterholzner *et al.*, 2011) and a predicted member of the VACV Bcl-2 family (Graham *et al.*, 2008; González & Esteban, 2010). Like other members of this family, C6 has an immunomodulatory function and inhibits the expression of IFN-β following stimulation of cells with multiple Toll-like receptor- and RIG-I-like-receptor-ligands by preventing the translocation of IFN regulatory factor (IRF)-3 into the nucleus (Unterholzner *et al.*, 2011). Accordingly, C6 was found to interact with TRAF family member-associated NF-κB activator (TANK), NF-κB-activating kinase-associated protein 1 (NAP1) and similar to NAP1 TANK-binding kinase 1 (TBK1) adaptor (SINTBAD). These are three TANK-binding kinase (TBK1)/inhibitor of κB kinase ϵ (IKKϵ) adaptor proteins with important roles in signal transduction and activation of IRF3 (Pomerantz & Baltimore, 1999; Fujita *et al.*, 2003; Ryzhakov & Randow, 2007). Although C6 had no effect on viral replication or spread *in vitro*, C6 promoted virulence because WR viruses not expressing C6 were attenuated in two models of murine infection (Unterholzner *et al.*, 2011). Given its role in innate immune modulation we decided to investigate whether C6 might affect VACV immunogenicity.

To investigate the potential effect of the removal of C6 on VACV immunogenicity, groups of five C57BL/6 mice were immunized intradermally (i.d.) in both ear pinnae (Tscharke & Smith, 1999; Tscharke *et al.*, 2002) with different doses of either a recombinant VACV strain WR engineered to lack C6 expression (vΔC6), a matching wild-type VACV (vC6WR) or a control virus where the *C6L* gene had been reinserted into the genome at its natural locus (vC6Rev) (Unterholzner *et al.*, 2011). Control mice were mock inoculated with PBS. A C6 deletion virus on the WR background was chosen for this study as WR is naturally virulent in mice, allowing assessment of both virulence and immunogenicity simultaneously, and these recombinant viruses had already been constructed (Unterholzner *et al.*, 2011). Previously, it was observed that the size of the lesion resulting from i.d. inoculation of mice with 10^4^ p.f.u. of vΔC6 was significantly smaller than an equivalent dose of a virus expressing C6 (vC6WR and vC6Rev) (Unterholzner *et al.*, 2011). A similar phenotype was also observed when mice were inoculated with 3×10^3^ p.f.u., with the lesion induced by vΔC6 being significantly smaller than that induced by the control viruses between 9 and 19 days post-infection (p.i.) (Fig. 1a). In contrast, when inoculated with 10^3^ p.f.u. the resulting lesions were similar in size (Fig. 1b), indicating that the observed attenuation of vΔC6 via this infection route is dose-dependent.

**Fig. 1.**
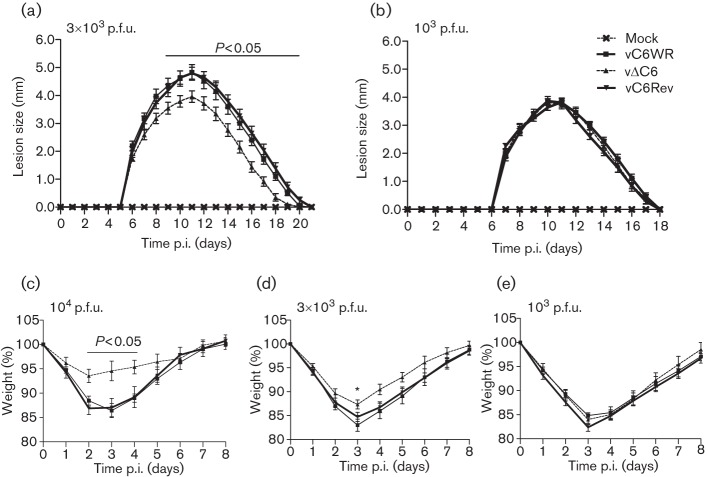
Vaccination of mice with vΔC6 provides enhanced protection against challenge despite its lower virulence. The lesions induced by i.d. inoculation (Tscharke & Smith, 1999) of groups of five C57BL/6 mice with the various C6 recombinant viruses at a dose of 3×10^3^ (a) or 10^3^ (b) p.f.u. in both ear pinnae were measured daily. Data are expressed as the mean lesion size (mm)±sem. Groups of five C57BL/6 mice that were vaccinated as described above with either 10^4^ (c), 3×10^3^ (d) or 10^3^ (e) p.f.u. were challenged i.n. (Williamson *et al.*, 1990) 1 month later with a lethal dose of wild-type WR (5×10^6^ p.f.u.) and the resulting weight loss was monitored daily. Data are expressed as the percentage±sem of the mean weight of the same group of animals on day 0. Significant differences between groups are shown, as determined using the Student’s *t*-test (*P<0.05). Data shown are representative of at least two experiments. All panels share the same figure legend, which is depicted in (b).

To test whether vΔC6 was more immunogenic than control viruses, mice immunized as above were challenged intranasally (i.n.) at 28 days p.i. (Williamson *et al.*, 1990) with a lethal dose (at least 100×LD_50_) of wild-type WR (5×10^6^ p.f.u.). All three vaccination doses provided protection against this lethal challenge; however, at two of the doses tested (10^4^ p.f.u., Fig. 1c and 3×10^3^ p.f.u., Fig. 1d) the weight loss observed for the vΔC6-vaccinated animals was significantly less than control virus-vaccinated animals. The greatest difference in protection was observed following immunization with 10^4^ p.f.u. per ear, with vΔC6-vaccinated mice displaying a maximum mean weight loss of 6.4 %, compared with 13.6 % after immunization with control viruses (Fig. 1c). A modest difference in weight loss was also observed following immunization with 3×10^3^ p.f.u. per ear, and this was statistically significant at 3 days post-challenge, corresponding with the time of maximum weight loss (Fig. 1d). In contrast, no difference in protection was observed with the 10^3^ p.f.u. per ear vaccination dose (Fig. 1e). Taken together these data indicated that vaccination of mice with vΔC6 provided better protection against subsequent VACV infection.

To understand the immunological basis of the enhanced protection provided by vΔC6, serological analysis was performed 1 month post-vaccination. The binding of serum antibodies to VACV-specific epitopes was assessed by ELISA, using plates that had been coated with a whole-cell lysate prepared from VACV-infected cells (Law *et al.*, 2005; Pütz *et al.*, 2006). Furthermore, the neutralization capacity of circulating antibodies was assessed by a plaque-reduction neutralization assay specific to the intracellular mature virion (IMV) form of VACV (Pütz *et al.*, 2006). Whereas the end-point antibody titre was equivalent between the groups of vaccinated animals (Fig. 2a), the dilution of antibody that provided 50 % neutralization (ND_50_) of IMV was lower in the vΔC6-vaccinated mice, indicating perturbation of the humoral response by this virus (Fig. 2b). Interestingly, a lower antibody neutralization capacity has also been observed with a recombinant WR virus lacking Bcl-2 family member K7 (unpublished data), an intracellular inhibitor of both NF-κB and IRF3 activation (Schröder *et al.*, 2008). Taken together these data indicated that the enhanced protection observed with vΔC6 was unlikely to be attributable to altered antibody responses.

**Fig. 2.**
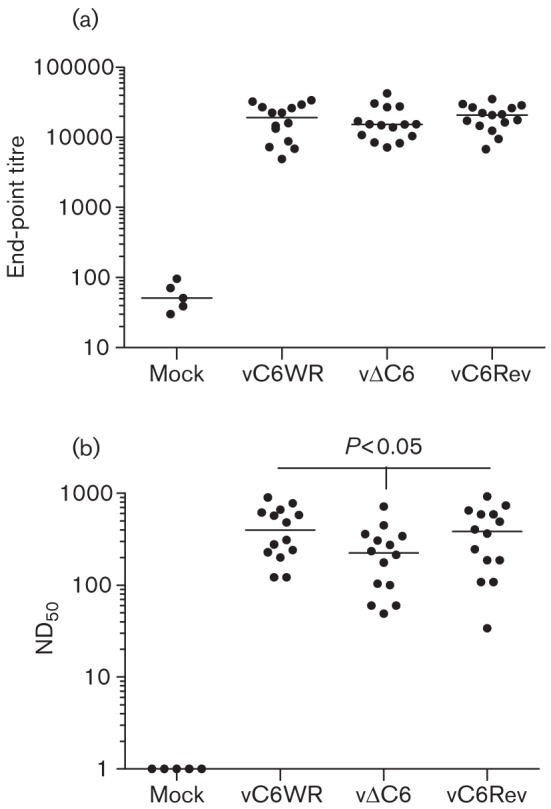
Humoral responses 1 month post-vaccination. Antibody end-point titres against VACV proteins (a) were determined by ELISA (Law *et al.*, 2005) from the serum of groups of five C57BL/6 mice that were vaccinated with 10^4^ p.f.u., or mock-vaccinated with PBS in both ear pinnae. End-point titres were defined as the reciprocal serum dilution giving twice the optical density obtained from BSA. A control serum from a mouse immunized with VACV was used to normalize end-point titres between ELISA plates (Pütz *et al.*, 2006). The neutralization capacity of antibodies in the serum of the animals described above was assessed by plaque-reduction neutralization (b) against VACV strain WR intracellular mature virus that had been purified by sucrose-density-gradient centrifugation (Pütz *et al.*, 2006). ND_50_ values were defined as the reciprocal of the dilution of serum giving a 50 % reduction in plaque number. For the VACV-vaccinated animals, data from three separate experiments were pooled. The median value for each population is represented by a horizontal black bar. Significant differences between groups are shown, as determined using the Mann–Whitney test.

To test whether vΔC6 was a better vaccine due to enhanced T-cell responses, a chromium release cytotoxicity assay was performed (Clark *et al.*, 2006). Spleens were harvested from mice 1 month post-vaccination, and splenocytes were prepared and incubated with VACV-infected EL-4 target cells that had been loaded with ^51^Cr. The percentage specific chromium release was higher using cells from vΔC6-vaccinated animals at effector-to-target ratios of 25 : 1, 50 : 1 and 100 : 1, and this was statistically significant at the latter ratio (Fig. 3a). The total number of CD4^+^ and CD8^+^ T-cells in the spleen of vaccinated animals at this time point was equivalent between the various groups of mice (data not shown). To assess whether the enhanced cytotoxic activity of T-cells correlated with enhanced release of IFN-γ, an enzyme-linked immunosorbent spot (ELISPOT) assay was performed on suspensions of splenocytes isolated at 1 month post-vaccination (Clark *et al.*, 2006). IFN-γ release by T-cells in response to VACV-infected EL-4 cells, or the CD8^+^ and C57BL/6-specific VACV peptide B8_20–27_ (Tscharke *et al.*, 2005) was not different between the groups of vaccinated animals (Fig. 3b). Mock-infected EL-4 cells and a CD8^+^ VACV peptide specific for BALB/c mice, E3_140–148_ (Tscharke *et al.*, 2006), were used as negative controls and IFN-γ release in response to these stimuli was minimal, as expected. Furthermore, no difference in the number of IFN-γ or tumour necrosis factor alpha (TNF-α)-secreting CD8^+^ cells from the spleens of vaccinated animals at 1 month post-vaccination was observed by intracellular cytokine staining (data not shown). Together, these data indicated that the VACV-specific cytotoxic T-cell activity 1 month post-vaccination was higher in the spleens of vΔC6-vaccinated mice, but a correlation with increased IFN-γ and/or TNF-α release at the dose tested was not found.

**Fig. 3.**
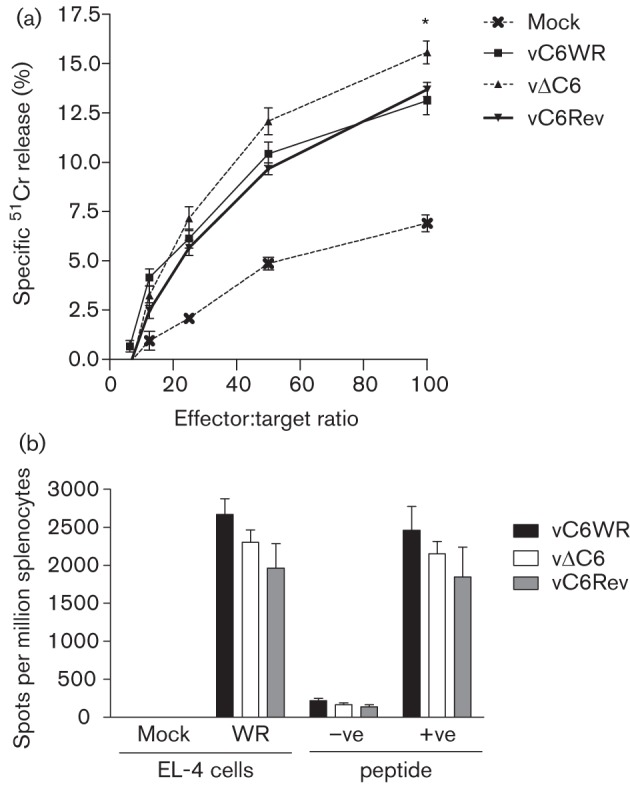
CD8^+^ T-cell responses 1 month post-vaccination. The cytolytic activity of T-cells derived from the spleens of groups of five C57BL/6 mice that had been vaccinated with 3×10^3^ p.f.u. of the indicated viruses, or mock vaccinated with PBS in both ear pinnae, was determined by ^51^Cr release assay (a) (Clark *et al.*, 2006). Cytolytic activity of splenic T-cells was assayed against VACV strain WR-infected EL-4 target cells and is presented as the mean percentage±sem. IFN-γ release by splenic T-cells of mice vaccinated as described above was assessed by ELISPOT assay (b) (Clark *et al.*, 2006). T-cells were stimulated to release IFN-γ *ex vivo* by incubation with WR-infected EL-4 target cells or the C57BL/6- and CD8^+^-specific B8_20–27_ peptide (+ve) (Tscharke *et al.*, 2005). As a control, T-cells were also stimulated with mock-infected EL-4 cells or the BALB/c-specific E3_140–148_ peptide (–ve) (Tscharke *et al.*, 2006). Data are presented as the mean number of spots per million splenocytes±sem. Significant differences between data obtained for vΔC6 from both vC6WR and vC6Rev are indicated, as analysed by the Student’s *t*-test (*P<0.05). Data are representative of at least two experiments.

In this study, the vaccine efficacy of vΔC6 was compared to control viruses expressing C6 at three different vaccination doses. Interestingly, where vΔC6 induced a smaller lesion than control viruses during the primary inoculation (i.e. 10^4^ and 3×10^3^ p.f.u.), there was also lower post-challenge weight loss (Fig. 1). On the other hand at an inoculation dose of 10^3^ p.f.u., where no difference in lesion size was observed between the three viruses, a difference in weight loss upon challenge was also not observed. Even VACV mutants that have increased virulence in the i.d. model can sometimes induce enhanced protection upon challenge. For instance, a VACV lacking the chemokine-binding protein A41 induced a larger lesion than WT VACV and yet provided enhanced protection (Clark *et al.*, 2006). Equally the removal of an immune modulator does not always lead to enhanced protection upon challenge, as exemplified by a recombinant VACV lacking protein C16 (Fahy *et al.*, 2008). Understanding why the removal of certain immune modulators leads to enhanced immunogenicity and why others do not warrants further investigation. Previously, it was demonstrated that a candidate HIV vaccine vector based on a recombinant MVA lacking C6 (MVA-B ΔC6) induced enhanced T-cell and antibody responses to the HIV antigens encoded by MVA-B compared with WT MVA-B (García-Arriaza *et al.*, 2011). However, whether this translates into enhanced protection against HIV infection remains to be determined (García-Arriaza *et al.*, 2011). Here, we show that removing C6 from VACV WR enhanced VACV-specific cytotoxic T-cell responses and resulted in a more efficacious vaccine that provided better protection against challenge.

In summary, data presented demonstrate that VACV strain WR lacking the expression of C6 was more immunogenic than control viruses, providing better protection against challenge with a lethal dose of VACV, despite being attenuated. This enhanced immunogenicity was not attributed to humoral responses, but instead correlated with enhanced VACV-specific cytotoxic T-cell activity 1 month post-vaccination. These data demonstrate that the removal of an intracellular innate immune modulatory protein can impact on the ensuing adaptive and memory immune responses. Fully understanding the mechanisms of how modulation of the innate response affects memory immunity is an important and interesting challenge for the future and will impact vaccine design.
